# Supplementary material for: Upcycling Glass Waste into Porous Microspheres for Wastewater Treatment Applications: Efficacy of Dye Removal
Source: Materials (Basel). 2022 Aug 23;15(17):5809. doi: 10.3390/ma15175809 (PMC9457513; doi:10.3390/ma15175809)
Supplement: Supplementary file 1 [file materials-15-05809-s001.zip › materials-1831999-supplementary.pdf]

Supplementary Section

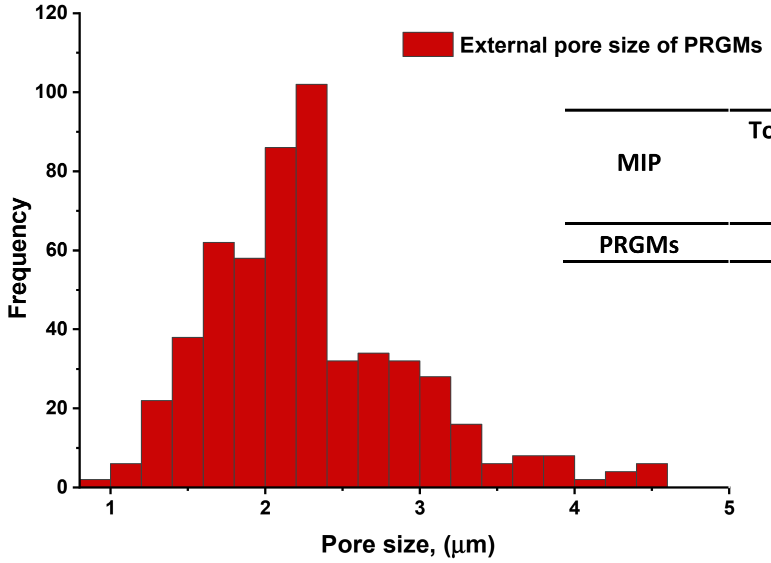

MIP	Total Intrusion Volume cm^3/g	Total Pore Area cm^2/g	Porosity %
PRGMs	0.84	8.6	69

Figure S1: Surface pore determination using SEM assisted Image J analysis with inset showing mercury porosimetry analysis of internal pores of PRGMs.

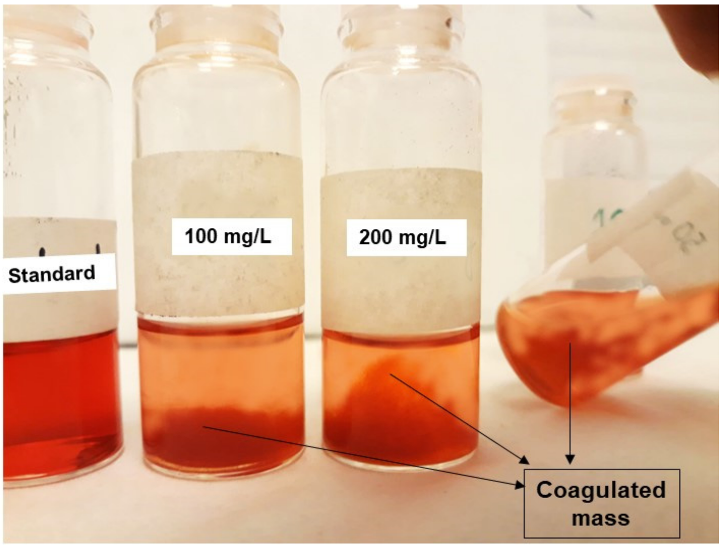

Figure S2: Effect of dye concentration on adsorption into W-PRGMs for AR88 (PRGMs dose= 10 g/L with pH 2.5 and temperature of 22 ± 2 ℃)

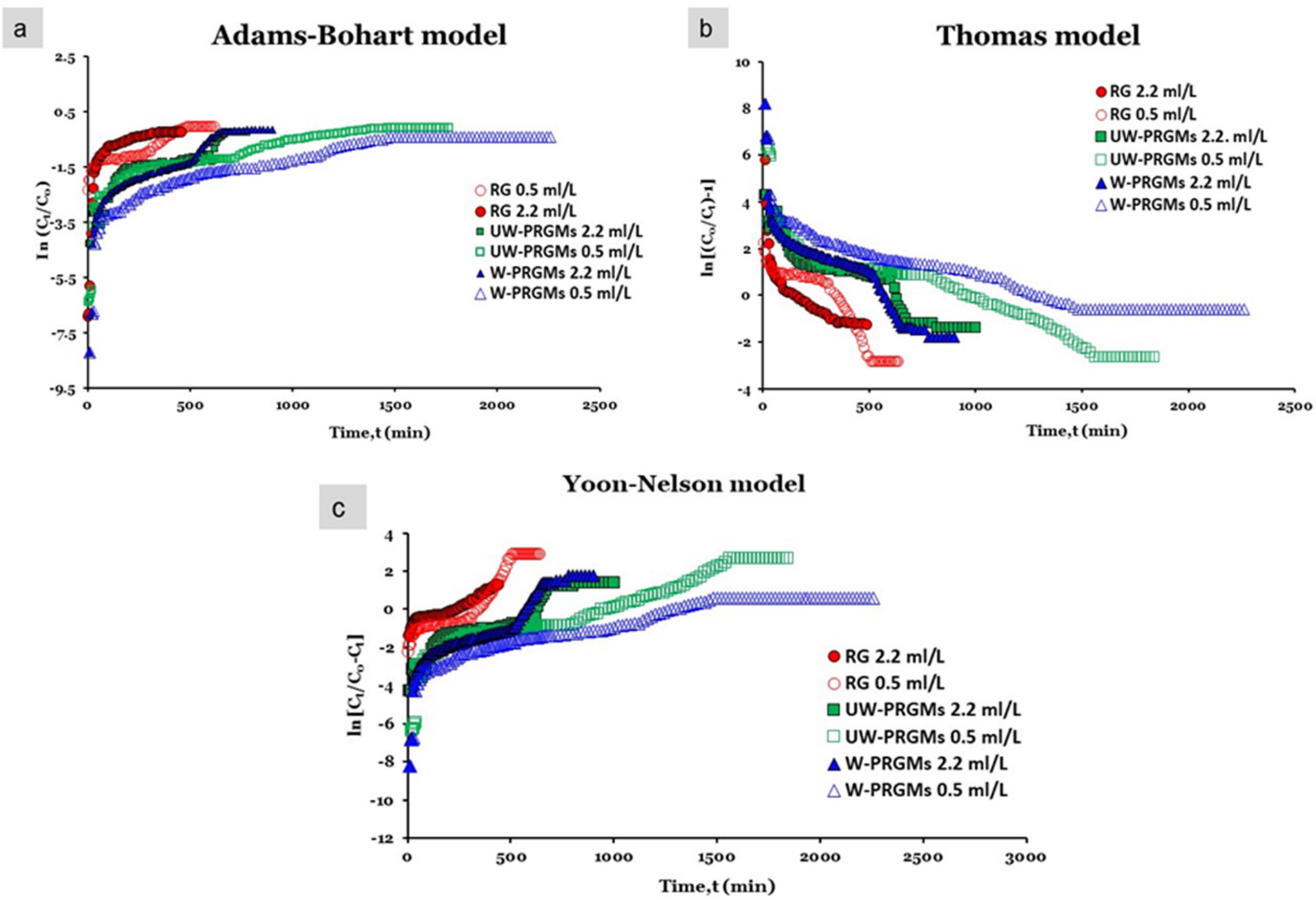

Figure S3: a) Adams-Bohart model, b) Thomas model, and c) Yoon-Nelson model at different flowrates (C_0= 100mg/L).

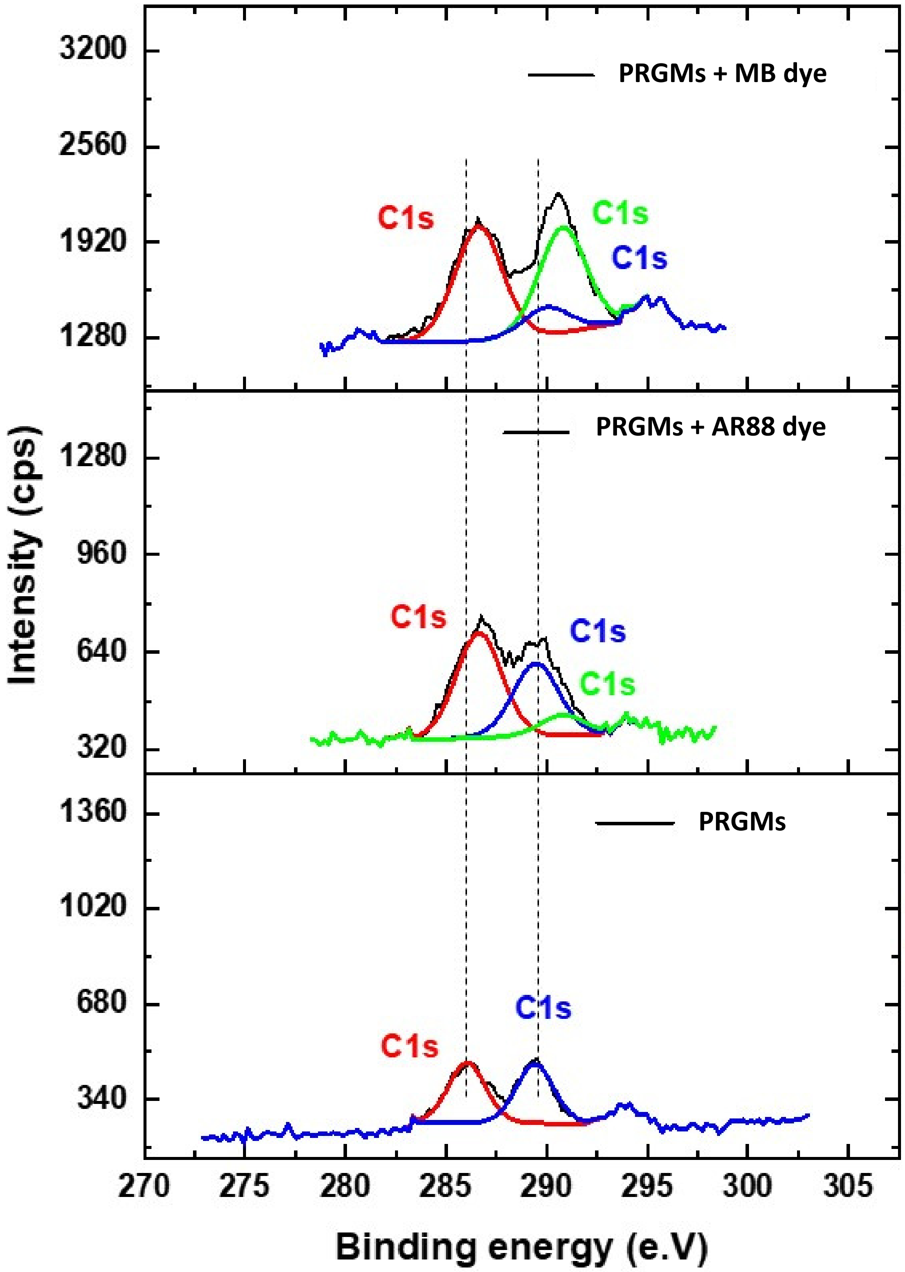

Peak	Assignments	B.E (eV)
C 1s	C-C/ C-H	286.7
C 1s	CO_3^{2-}	289.7
C 1s	π-π*	290.8

Peak	Assignments	B.E (eV)
C 1s	C-C/ C-H	286.7
C 1s	CO_3^{2-}	289.4
C 1s	π-π*	290.8

Peak	Assignments	B.E (eV)
C 1s	C-C/ C-H	286
C 1s	CO_3^{2-}	289.4

Figure S4: High resolution XPS spectra of C 1s for PRGMs before and after AR88 and MB adsorption.

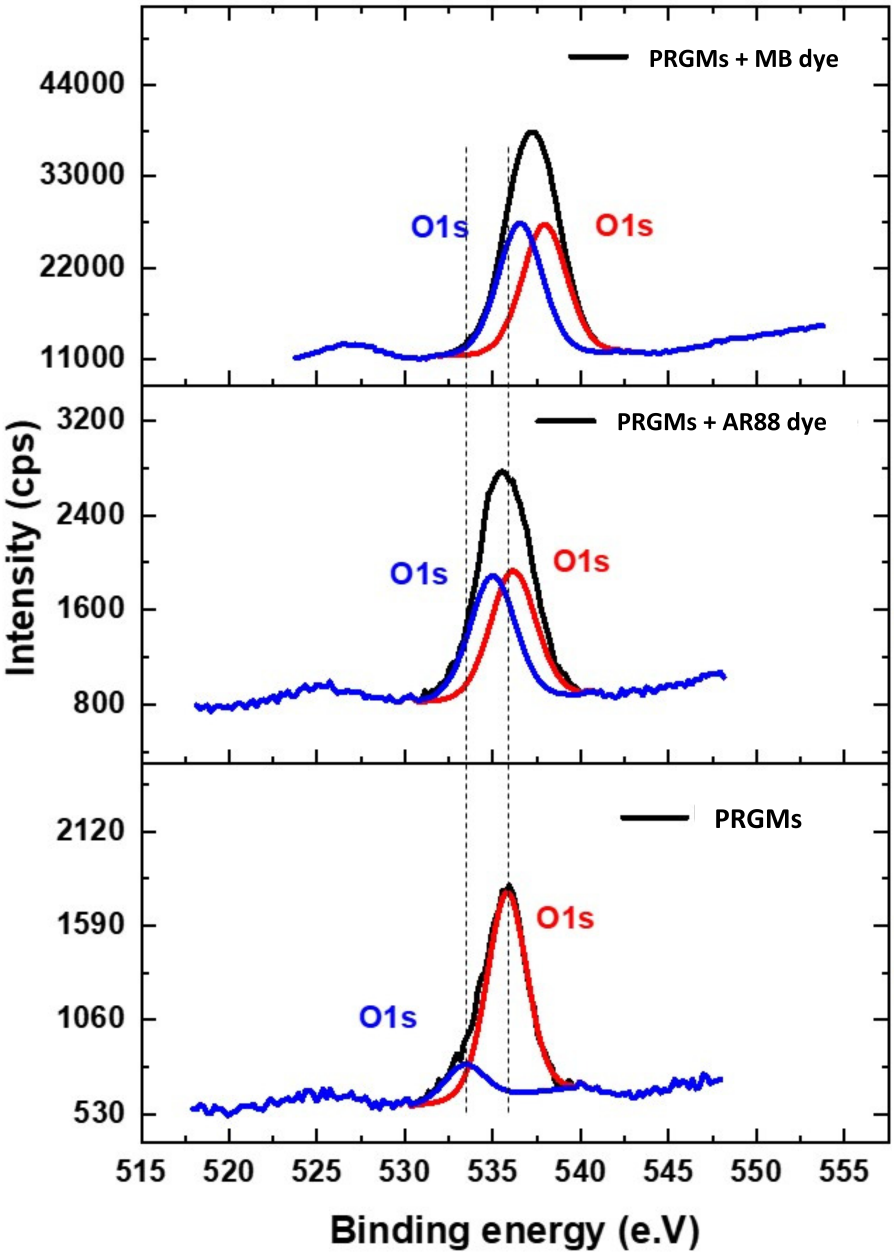

Peak	Assignments	B.E (eV)
O 1s	NBO	536.5
O 1s	BO	537.9

Peak	Assignments	B.E (eV)
O 1s	NBO	534.8
O 1s	BO	536.2

Peak	Assignments	B.E (eV)
O 1s	NBO	533.3
O 1s	BO	535.8

Figure S5: High resolution XPS spectra of O 1s for PRGMs before and after AR88 and MB adsorption.

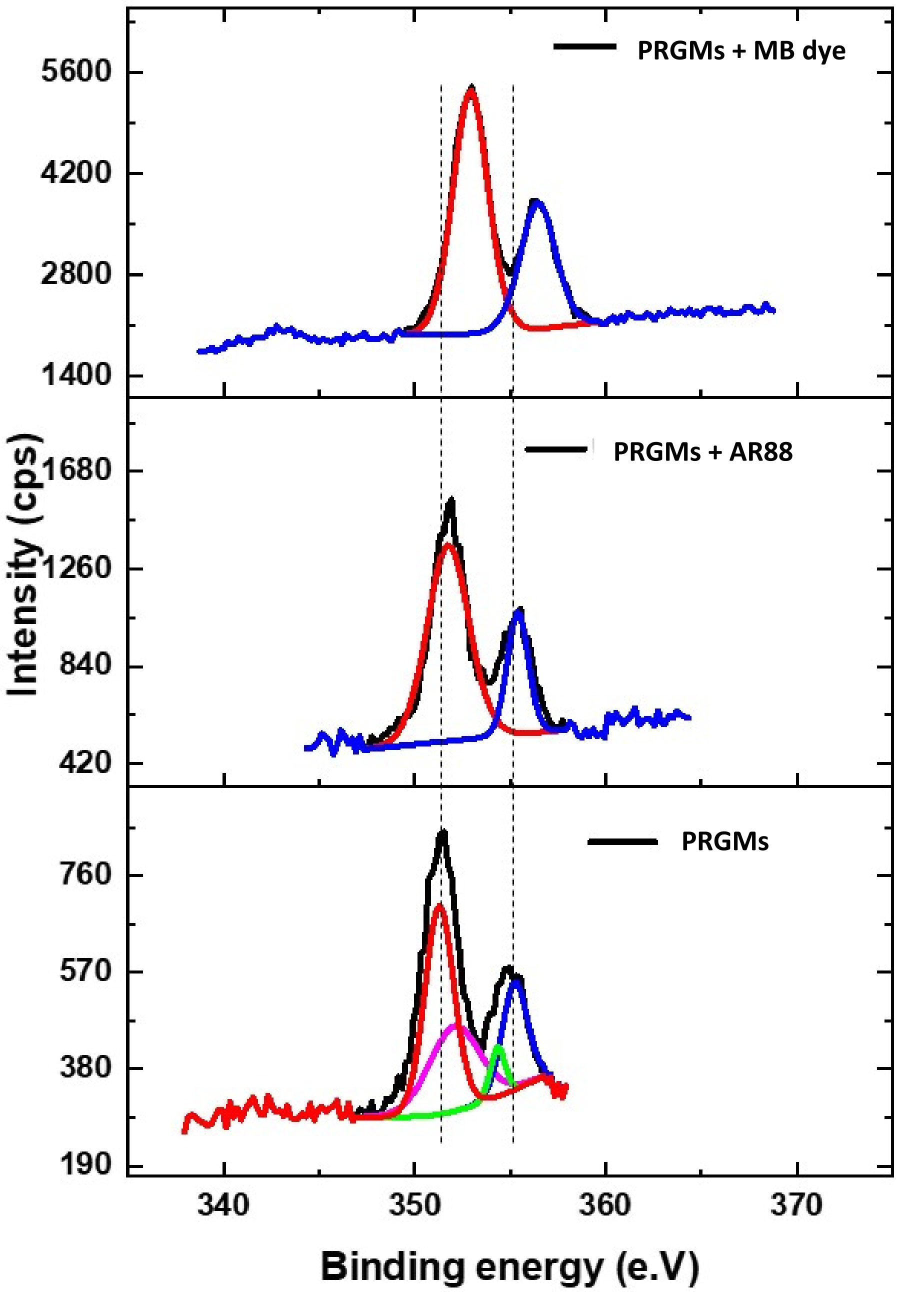

Peak	Assignmei	B.E (eV)
Ca $2P_{3/2}$	Ca in RG	352.9
Ca $2P_{1/2}$	Ca in RG	356.4

Peak	Assignments	B.E (eV)
Ca $2P_{3/2}$	Ca in RG	351.5
Ca $2P_{1/2}$	Ca in RG	355.5

Peak	Assignments	B.E (eV)
Ca $2P_{3/2}$	Ca in $CaCO_3$	351.3
Ca $2P_{3/3}$	Ca in RG	352.4
Ca $2P_{1/2}$	Ca in $CaCO_3$	354.3
Ca $2P_{1/3}$	Ca in RG	355.3

Figure S6: High resolution XPS spectra of Ca 2p for PRGMs before and after AR88 and MB adsorption.

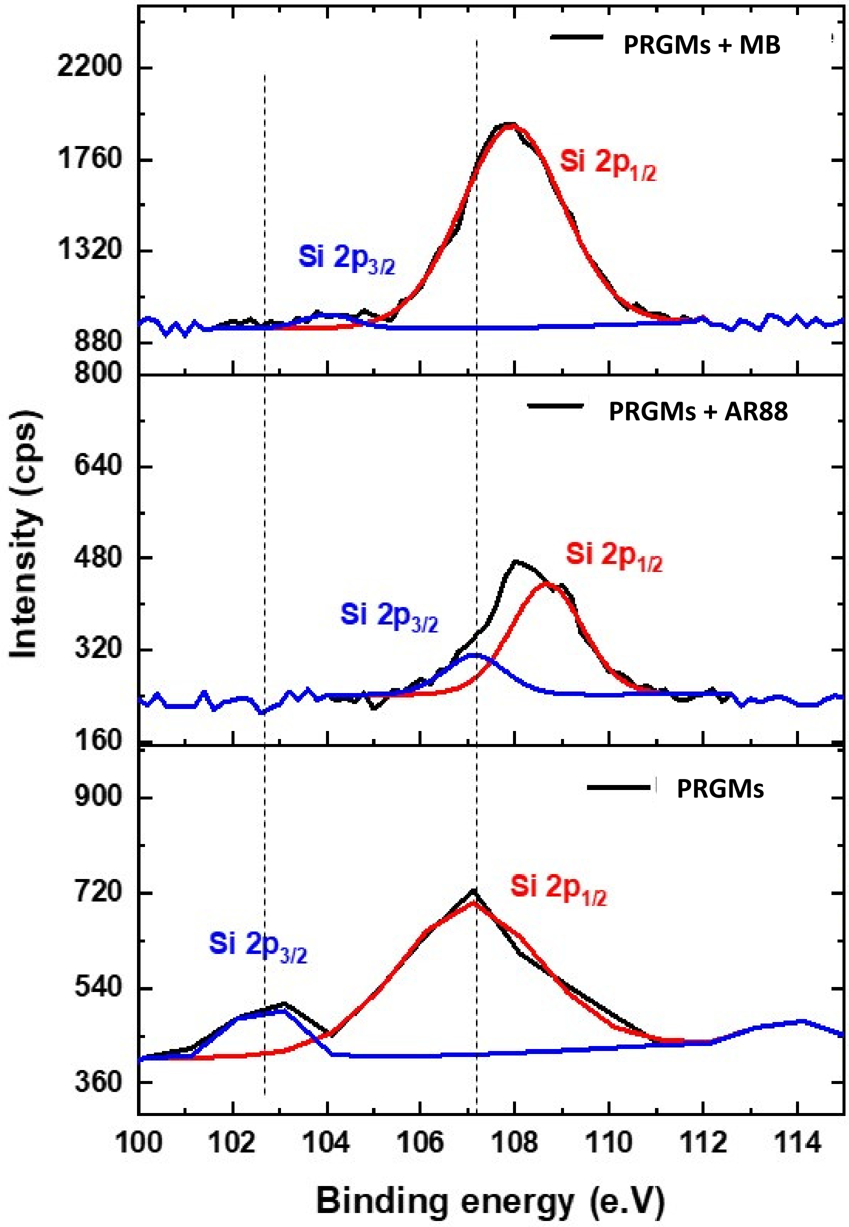

Peak	Assignmer	B.E (eV)
Si $2P_{3/2}$	Si in RG	104.9
Si $2P_{1/2}$	Si in RG	107.9

Peak	Assignments	B.E (eV)
Si $2P_{3/2}$	Si in RG	107.2
Si $2P_{1/2}$	Si in RG	108.7

Peak	Assignments	B.E (eV)
Si $2P_{3/2}$	Si in RG	102.7
Si $2P_{1/2}$	Si in RG	107

Figure S7: High resolution XPS spectra of Si 2p for PRGMs before and after AR88 and MB adsorption.
